# Pollock oil supplementation modulates hyperlipidemia and ameliorates hepatic steatosis in mice fed a high-fat diet

**DOI:** 10.1186/1476-511X-10-189

**Published:** 2011-10-25

**Authors:** Zhi-Hong Yang, Hiroko Miyahara, Jiro Takeo, Akimasa Hatanaka, Masashi Katayama

**Affiliations:** 1 Central Research Laboratory, Tokyo Innovation Center, Nippon Suisan Kaisha, Ltd., 32-3 Nanakuni 1 Chome Hachioji, Tokyo 192-0991, Japan

**Keywords:** Pollock oil, n-3 PUFA, MUFA, hyperlipidemia, hepatic steatosis, adipokines

## Abstract

**Background:**

Hyperlipidemia associated with obesity is closely related to the development of atherosclerosis. Both n-3 polyunsaturated fatty acids (PUFAs) and long-chain monounsaturated fatty acids (MUFAs; i.e., C20:1 and C22:1 isomers) supplementation modulate risk factors for metabolic syndrome via multiple mechanisms, including the restoration of impaired lipid metabolism. We therefore examined the effects of pollock oil, which contains a considerable amount of n-3 PUFAs as well as long-chain MUFAs, on plasma hyperlipidemia and hepatic steatosis in diet-induced obese mice.

**Methods:**

Male C57BL/6J mice (24-26 g) were divided into two groups (n = 10/group) and were fed a high-fat diet containing 32% lard (control group) or 17% lard plus 15% pollock oil (experimental group) for 6 weeks. For both groups, fat comprised 60% of the total caloric intake.

**Results:**

Although body and liver masses for the two groups did not differ significantly, hepatic lipids concentrations (triglycerides and total cholesterols) were lower (*P *< 0.05) after pollock oil ingestion. After 2 weeks on the specified diets, plasma lipid levels (total cholesterol, LDL cholesterol, and triglycerides) significantly decreased (*P *< 0.05) in the experimental group compared with the control group, although plasma HDL cholesterol levels did not differ. At the end of 6 weeks, plasma adiponectin levels increased (*P *< 0.05), whereas plasma resistin and leptin levels decreased (*P *< 0.05) in the experimental mice. Increased levels of long-chain MUFAs and n-3 PUFAs in plasma, liver and adipose tissue by ingesting pollock oil were possibly correlated to these favorable changes. Expression of hepatic genes involved in cholesterol metabolism (*SREBP2*, *HMGCR*, and *ApoB*) and lipogenesis (*SREPB1c*, *SCD-1*, *FAS*, and *Acac*α) was suppressed in the experimental group, and may have favorably affected hyperlipidemia and hepatic steatosis induced by the high-fat diet.

**Conclusions:**

We demonstrated that pollock oil supplementation effectively improved hyperlipidemia, attenuated hepatic steatosis, and downregulated the express of hepatic genes involved in cholesterol and lipid metabolism in mice with diet-induced obesity.

## Background

Hyperlipidemia, a medical condition characterized by increased blood levels of lipids including cholesterol and triglycerides, is a critical component of metabolism syndrome as well as a possible predisposing factor for atherosclerosis, a leading cause of death worldwide [1,2]. Lipids that accumulate in the arterial wall as a consequence of hyperlipidemia are oxidized and attract inflammatory monocytes, which differentiate into macrophages that take up the oxidized lipid. Oxidation of low-density lipoproteins (LDLs) in the arterial wall is a major and physiologically relevant mechanism for the pathogenesis of atherosclerosis, and the presence of lipid-loaded macrophage foam cells in the artery intima is a predictor for the development of atherosclerotic lesions. A close relationship exists between dietary fats and dyslipidemia-related events [3]. Although an increased intake of saturated fatty acids is pathogenic for coronary heart disease, numerous studies have demonstrated a protective effect of n-3 polyunsaturated fatty acids (PUFAs) through a variety of mechanisms, including reduction of triglycerides and very-low-density lipoproteins [4]. In addition, we have shown that marine-derived long-chain monounsaturated fatty acids (MUFAs) (i.e., C20:1 and C22:1 isomers) modulate metabolic syndrome by restoring impaired glucose and lipid metabolism [5]. Therefore, fish oils that are rich in both n-3 PUFAs and long-chain MUFAs may help alleviate hypercholesterolemia and hypertriacylglyceridemia.

Alaska pollock (*Theragra chalcogramma*) is a North Pacific species of the cod family, Gadidae. Pollock oil contains considerable amounts of n-3 PUFAs and long-chain MUFAs [6]. The Alaska pollock fishing industry is the largest in the United States and one of the largest in the world. In recent years, pollock fishing has accounted for ~30% of all U.S. seafood landings by mass [7]. Although pollock oil is used in both the food and feed industries [8], little is known about the relationship between dietary pollock oil and hyperlipidemia. Given the health benefits of n-3 PUFAs and long-chain MUFAs, we examined the effect of dietary pollock oil on hyperlipidemia in mice with diet-induced dyslipidemia.

## Methods

### Measurement of fatty acid composition of dietary oils

Cameria lard was purchased from Romi Smilfood B. V. (Heerenveen, Netherlands). Pollock oil was obtained from Nippon Suisan Kaisha, Ltd. (Tokyo, Japan) and refined with silica gel and activated clays and then steam-distillation deodorized. All standard and extracted lipids were stored at -20°C until used. Fatty acid compositions of the dietary fats (Table 1) were determined after methylation of samples with 14% (w/v) boron trifluoride/methanol (Sigma Chemical Co., St. Louis, USA.) at 80°C for 30 min. The resulting fatty acid methyl esters were quantified by gas chromatography using an Agilent 6890N Network Gas Chromatograph System (Agilent Technologies Japan, Ltd., Tokyo, Japan) equipped with a split injector, FID detector, and fused silica capillary column (DB-WAX, 30 m × 0.25 mm I.D. × 0.25 μm film thickness, J & W Scientific, Agilent Technologies). Methyl esters were identified by comparison of retention times to those of fatty acid methyl ester standards (Nu-Chek Prep, Inc., Elysian, MN, USA). Pollock oil contains considerable levels of long-chain MUFAs and n-3 PUFAs (C20:1 as well as C22:1 isomers and n-3 PUFAs combined: ~ 45%).

**Table 1:** Fatty acid composition of dietary fats (%)


**Fatty acid**	**Lard **	**Pollock oil**

C14:0	1.5	4.9
C16:0	25.4	9.8
C16:1	2.4	6.1
C18:0	5.9	1.7
C18:1	40.6	14.3
C18:2 n-6	10.8	1.3
C18:3 n-3	1.0	1.1
C20:1 n-9	0.8	9.1
C20:1 n-7	ND	3.3
C22:1 n-11	ND	12.3
C22:1 n-9	ND	1.6
C20:5 n-3	0.02	10.3
C22:5 n-3	0.1	1.2
C22:6 n-3	0.03	7.9

Values correspond to mean of three separate samples processed independently.

ND: Not detected.

### Animals and diets

The Institutional Animal Care and Use Committee at Nihon Bioresearch Inc. (Gifu, Japan) approved this study. Male C57BL/6J mice (5 weeks old) were obtained from Charles River Laboratories Japan Inc. (Yokohama, Japan) and housed at Nihon Bioresearch at 23 ± 1°C under a 12/12 h light-dark cycle. The animals were provided free access to water and standard mouse chow CRF-1 (Oriental Yeast Co. Ltd., Tokyo, Japan) for a 1-week acclimatization period.

After acclimatization, mice were randomly assigned to one of two groups for the 6-week feeding experiment. The control group (n = 10) was fed a high-fat diet containing 32% lard (D12492 Rodent Diet with 60 kcal% Fat; Research Diets, Inc., NJ, USA) and the experimental group was fed a diet supplemented with pollock oil (17% lard plus 15% pollock oil). To control for total fat intake, the total fat content in both diets corresponded to 60% of the caloric intake. The compositions of the diets are listed in Table 2. Body mass and food intake were monitored throughout the study. At the end of the intervention period, mice were anesthetized with 4% sodium pentobarbital (Dainippon Sumitomo Pharma, Osaka, Japan), and blood was collected by abdominal vein puncture. Plasma was obtained by centrifugation at 1000 g for 15 min and stored at -80°C until analyses. Vital organs were removed and weighed after a short wash in cold phosphate-buffered saline, pH 7.4. Mesenteric white adipose tissue (WAT) and Livers were kept at -80°C for the further lipid extraction and quantitative polymerase chain reaction (QPCR) analysis.

**Table 2:** Diet compositions


**Ingredient**	**Lard diet (g/100 g diet)**	**PO diet (g/100 g diet)**

Casein	25.8	25.8
l-Cysteine	0.4	0.4
Maltodextrin 10	16.2	16.2
Sucrose	8.9	8.9
Cellulose	6.5	6.5
Mineral mixture	1.3	1.3
Vitamin mixture	1.3	1.3
Choline bitartrate	0.3	0.3
Soybean oil	3.2	3.2
Lard	32	17
Pollock oil	--	15

PO diet: Pollock oil-supplemented diet.

### Lipid extraction and fatty acid analysis

The fatty acid compositions of plasma, liver and WAT in the C57BL/6J mice were determined as described before [5]. Lipids were extracted by homogenizing the tissue samples in a methanol/hexane solution (4:1 v/v) added with butylated hydroxytoluene (BHT, 50 μg/mL) as an antioxidant. The samples were methylated with acetyl chloride, and the fatty acid methyl esters were separated and quantified by gas chromatography. Identification of the methyl esters were made by comparison of retention times of standard fatty acids.

### Determination of plasma lipid levels

Blood samples were taken from the retro-orbital venous plexus of each mouse at the end of weeks 0, 2, 4, and 6. Plasma concentrations of triglyceride (TG), total cholesterol (TC), and high-density lipoprotein (HDL) cholesterol were measured using Triglycerol E-Test, Cholesterol E-Test, and HDL-cholesterol E-Test kits (Wako Pure Chemical Industries, Ltd., Osaka, Japan), respectively. The concentration of LDL cholesterol was calculated as [LDL cholesterol] = [TC] - [HDL cholesterol] - [TG] × 0.2.

### Determination of plasma adipokine levels

Plasma concentrations of adiponectin, resistin, and leptin were determined at the end of the 6-week period using the Mouse Adiponectin ELISA kit (Otsuka Pharmaceutical Co., Ltd., Tokyo, Japan), Mouse Resistin ELISA kit (Shibayagi Co. Ltd., Gunma, Japan), and Mouse Leptin ELISA kit (Morinaga Institute of Biological Science, Inc., Yokohama, Japan), respectively.

### Determination of hepatic lipid levels

Total hepatic lipids were extracted from liver samples as described [9]. Extracted lipids were dried under vacuum (Concentrator Plus 5305, Eppendorf Inc., NY, USA) and then dissolved in 2-propanol containing 10% (w/w) Triton X-100. Triglyceride and total cholesterol concentrations were determined using the above-mentioned commercial enzyme kits (Wako).

### Determination of mRNA expression by QPCR

Total RNA was isolated from liver samples using TRIzol reagent (Qiagen, Valencia, CA, USA) according to the manufacturer's protocol. First-strand cDNA was generated from total RNA (1 μg) using the PrimeScript II 1st strand cDNA Synthesis kit (TaKaRa Bio, Otsu, Japan). The resulting cDNA was used for QPCR amplification and specific sequence detection on an Applied Biosystems 7300 Real-Time PCR System (Life Technologies Ltd., Tokyo, Japan). The PCR cycling parameters were 30 s at 95°C; 40 cycles of 5 s at 95°C, 34 s at 60°C; and a final melting curve of 15 s at 95°C, 1 min at 60°C, 15 s at 95°C. Gene expression was scaled to the expression of the housekeeping gene encoding 18S ribosomal RNA. PCR reactions contained forward and reverse primers (10 μM each) and SYBR Premix Ex Taq (TaKaRa Bio). The targeted genes, their corresponding proteins, and the respective sense and antisense PCR primers were: *SREBP2 *(sterol regulatory element binding protein 2), 5'- TGGGCGATGAGCTGACTCT-3' and 5'- ACTGTAGCATCTCGTCGATGT-3'; *HMGCR *(3-hydroxy-3-methylglutaryl-coenzyme A reductase), 5'- TGTTCACCGGCAACAACAAGA-3' and 5'-CCGCGTTATCGTCAGGATGA-3'; *ApoB *(apolipoprotein B), 5'-TTGGCAAACTGCATAGCATCC-3' and 5'-TCAAATTGGGACTCTCCTTTAGC-3'; *ApoA *(apolipoprotein A), 5'- GGCACGTATGGCAGCAAGAT-3' and 5'-CCAAGGAGGAGGATTCAAACTG-3'; *SREBP1c *(sterol regulatory element binding protein 1c), 5'-GATGTGCGAACTGGACACAG-3' and 5'-CATAGGGGGCGTCAAACAG-3'; *SCD-1 *(stearoyl-coenzyme A desaturase-1), 5'-TTCTTGCGATACACTCTGGTGC-3' and 5'-CGGGATTGAATGTTCTTGTCGT-3'; *FAS *(fatty acid synthase), 5'-TTCTTGCGATACACTCTGGTGC-3' and 5'-CGGGATTGAATGTTCTTGTCGT-3'; *Acacα *(acetyl-coenzyme A carboxylase alpha), 5'-GATGAACCATCTCCGTTGGC-3' and 5'-CCCAATTATGAATCGGGAGTGC-3'.

### Statistical analysis

Results are expressed as mean ± standard error of the mean. Statistical differences between two groups were analyzed by Student's *t*-test and were considered significant at *P *< 0.05.

## Results

### Effect of pollock oil on body and organ mass

Table 3 lists body and vital organ masses for diet-induced obese C57BL/6J mice in the control (lard) and experimental (pollock oil) groups. After the 6-week feeding period, there were no significant differences in body, liver, white as well as brown adipose tissue, and skeleton muscle masses between the two groups.

**Table 3:** Food intake, body mass, and vital organ masses


	**Lard diet**	**PO diet**

Food intake (g/day)	2.5 ± 0.03	2.3 ± 0.04
Initial body mass (g)	25.5 ± 0.4	25.5 ± 0.5
Final body mass (g)	32.7 ± 1.6	31.6 ± 2.4
Organ masses (mg/g body mass)		
Liver	33.1 ± 0.7	33.6 ± 0.7
Epididymal WAT	50.9 ± 3.0	45.2 ± 3.9
Mesenteric WAT	15.4 ± 0.9	13.7 ± 1.1
Brown adipose tissue	3.3 ± 0.5	3.6 ± 0.3
Skeletal muscle	5.9 ± 0.4	5.9 ± 0.3

Each value represents the mean ± SE (n = 10). PO diet: Pollock oil-supplemented diet; WAT: white adipose tissue.

### Effect of pollock oil on plasma lipid levels

Compared to control, total cholesterol plasma levels for the experimental group were reduced by 27% (*P *< 0.01), 17% (*P *< 0.01), and 30% (*P *< 0.001) at the end of weeks 2, 4, and 6, respectively (Table 4). Pollock oil intake also significantly reduced plasma LDL cholesterol levels by 33% (*P *< 0.05), 23% (*P *< 0.01), and 38% (*P *< 0.001), and reduced plasma triglyceride levels by 40% (*P *< 0.01), 50% (*P *< 0.01), and 46% (*P *< 0.01) at the end of weeks 2, 4, and 6, respectively. No significant differences in plasma HDL cholesterol levels were detected.

**Table 4:** Plasma levels of total cholesterol, LDL cholesterol, HDL cholesterol, and triglycerides


**Lipids (mg/dL)**	**Week 0**	**Week 2**	**Week 4**	**Week 6**

**Total cholesterol**				
Lard diet	84.7 ± 7.1	154.5 ± 10.5	158.1 ± 4.5	161.5 ± 4.3
PO diet	80.8 ± 6.0	112.9 ± 3.7**	130.8 ± 5.7**	113.1 ± 2.7***
**LDL cholesterol**				
Lard diet	39.1 ± 7.0	83.5 ± 9.3	87.7 ± 3.4	78.5 ± 3.5
PO diet	38.1 ± 5.6	56.1 ± 3.1*	67.4 ± 5.4**	56.6 ± 2.5***
**HDL cholesterol**				
Lard diet	34.6 ± 2.0	54.4 ±2.8	51.4 ± 2.0	59.2 ± 2.5
PO diet	30.6 ± 2.4	47.3 ± 1.8	53.7 ± 2.7	43.7 ± 3.4
**Triglycerides**				
Lard diet	56.4 ± 4.4	80.0 ± 10.1	95.7 ± 12.9	118.7 ± 19.8
PO diet	60.6 ± 4.9	47.7 ± 6.7**	48.2 ± 6.8**	63.9 ± 5.7**

Each value represents the mean ± SE (n = 10). PO diet: Pollock oil-supplemented diet; LDL: low-density lipoprotein; HDL: high-density lipoprotein; *P < 0.05; **P < 0.01; ***P < 0.001.

### Effect of pollock oil on fatty acid compositions of plasma, liver and WAT

Plasma, liver and mesenteric WAT fatty acid compositions in the control and pollock oil group are shown in Table 5 and Table 6. Although total saturated fatty acid levels did not differ between the control and experimental group, pollock oil ingestion markedly (*P *< 0.05) increased long-chain MUFA (i.e., C20:1 and C22:1 isomers combined) levels 3-, 1.2- and 5-fold in plasma, liver and WAT, respectively. Intake of pollock oil also significantly (*P *< 0.05) increased total n-3 PUFA levels 3-, 2- and 7-fold in plasma, liver and WAT, respectively. In contrast, total n-6 PUFA levels were significantly (*P *< 0.05) decreased by 50%, 31% and 14% in plasma, liver and WAT, respectively in the pollock oil group as compared to the control.

**Table 5:** Fatty acid composition in plasma (%)


**Fatty acid**	**Lard diet**	**PO diet**

14:0	0.20 ± 0.02	0.29 ± 0.02
16:0	13.95 ± 0.33	11.24 ± 1.17
18:0	8.68 ± 0.24	6.80 ± 0.86
SAF	22.84 ± 0.47	18.33 ± 2.03
12:1	23.68 ± 0.90	24.71 ± 1.84
16:1	0.69 ± 0.10	0.79 ± 0.08
18:1	9.23 ± 0.37	7.77 ± 0.52*
20:1 n-9	0.18 ± 0.01	0.24 ± 0.02*
20:1 n-7	ND	0.22 ± 0.02***
22:1 n-11	ND	0.17 ± 0.02**
22:1 n-9	ND	0.01 ± 0
MUFA	33.77 ± 0.97	33.91 ± 2.42
18:2 n-6	17.04 ± 0.25	10.81 ± 1.13**
18:3 n-6	0.26 ± 0.01	0.08 ± 0.01***
20:2 n-6	0.13 ± 0	0.07 ± 0.01*
20:3 n-6	0.89 ± 0.07	0.29 ± 0.04***
20:4 n-6	14.03 ± 0.57	3.61 ± 0.36***
22:4 n-6	0.02 ± 0	0.02 ± 0
n-6 PUFA	32.37 ± 0.38	14.87 ± 1.54***
18:3 n-3	0.18 ± 0.02	0.16 ± 0.02
20:5 n-3	0.40 ± 0.03	6.32 ± 0.81**
22:5 n-3	0.32 ± 0.07	4.71 ± 1.76**
22:6 n-3	5.11 ± 0.11	11.62 ± 2.04**
n-3 PUFA	6.01 ± 0.11	22.85 ± 2.18**

Each value represents the mean ± SE (n = 10). ND: Not detected; PO diet: Pollock oil-supplemented diet; SAF: saturated fatty acids; MUFA: monounsaturated fatty acids; PUFA: polyunsaturated fatty acids; *P < 0.05; **P < 0.01; ***P < 0.001.

**Table 6:** Fatty acid composition in liver and mesenteric WAT (%)


**Fatty acid**	**Liver**		Mesenteric WAT	
	
	Lard diet	PO diet	Lard diet	PO diet

14:0	0.29 ± 0.01	0.30 ± 0.01	1.03 ± 0.03	1.85 ± 0.03*
16:0	21.47 ± 0.13	20.18 ± 0.24***	20.43 ± 0.11	19.43 ± 0.23**
18:0	8.09 ± 0.13	10.06 ± 0.21***	4.39 ± 0.11	5.02 ± 0.15**
SAF	29.85 ± 0.15	30.55 ± 0.17	25.85 ± 0.15	26.29 ± 0.22
12:1	7.37 ± 0.29	7.87 ± 0.28	2.11 ± 0.10	1.98 ± 0.09
16:1	1.46 ± 0.05	1.25 ± 0.07*	4.05 ± 0.21	3.94 ± 0.17
18:1	22.55 ± 0.35	13.58 ± 0.47***	45.71 ± 0.15	37.42 ± 0.33***
20:1 n-9	0.51 ± 0.02	0.60 ± 0.02**	0.85 ± 0.03	3.12 ± 0.13***
20:1 n-7	ND	0.41 ± 0.01***	0.15 ± 0	1.55 ± 0.05***
22:1 n-11	ND	0.19 ± 0.01***	0.05 ± 0	1.37 ± 0.08***
22:1 n-9	0.06 ± 0	0.06 ± 0.01	0.04 ± 0	0.25 ± 0.01**
MUFA	32.05 ± 0.32	23.86 ± 0.51***	52.95 ± 0.23	49.63 ± 0.51**
18:2 n-6	14.13 ± 0.28	11.89 ± 0.16***	14.47 ± 0.17	12.70 ± 0.28***
18:3 n-6	0.29 ± 0.01	0.10 ± 0.03***	0.07 ± 0	0.06 ± 0.01
20:2 n-6	0.22 ± 0	0.12 ± 0***	0.27 ± 0	0.17 ± 0.03*
20:3 n-6	0.86 ± 0.04	0.47 ± 0.03***	0.12 ± 0	0.06 ± 0***
20:4 n-6	9.31 ± 0.15	4.66 ± 0.09***	0.24 ± 0.01	0.15 ± 0***
22:4 n-6	0.31 ± 0.01	0.03 ± 0***	0.07 ± 0	0.03 ± 0***
n-6 PUFA	25.11 ± 0.18	17.26 ± 0.18***	15.23 ± 0.17	13.17 ± 0.28***
18:3 n-3	0.45 ± 0.02	0.56 ± 0.03**	0.07 ± 0	0.96 ± 0.04***
20:5 n-3	0.29 ± 0.01	4.84 ± 0.17***	0.10 ± 0	0.73 ± 0.05***
22:5 n-3	0.51 ± 0.01	1.66 ± 0.07***	0.11 ± 0	0.44 ± 0.01***
22:6 n-3	6.32 ± 0.12	15.36 ± 0.26***	0.18 ± 0	1.42 ± 0.04***
n-3 PUFA	7.55 ± 0.12	22.42 ± 0.18***	0.45 ± 0.01	3.54 ± 0.12***

Each value represents the mean ± SE (n = 10). ND: Not detected; WAT: white adipose tissue; PO diet: Pollock oil-supplemented diet; SAF: saturated fatty acids; MUFA: monounsaturated fatty acids; PUFA: polyunsaturated fatty acids; *P < 0.05; **P < 0.01; ***P < 0.001.

### Effect of pollock oil on plasma adipocytokine levels

**Figure 1:**
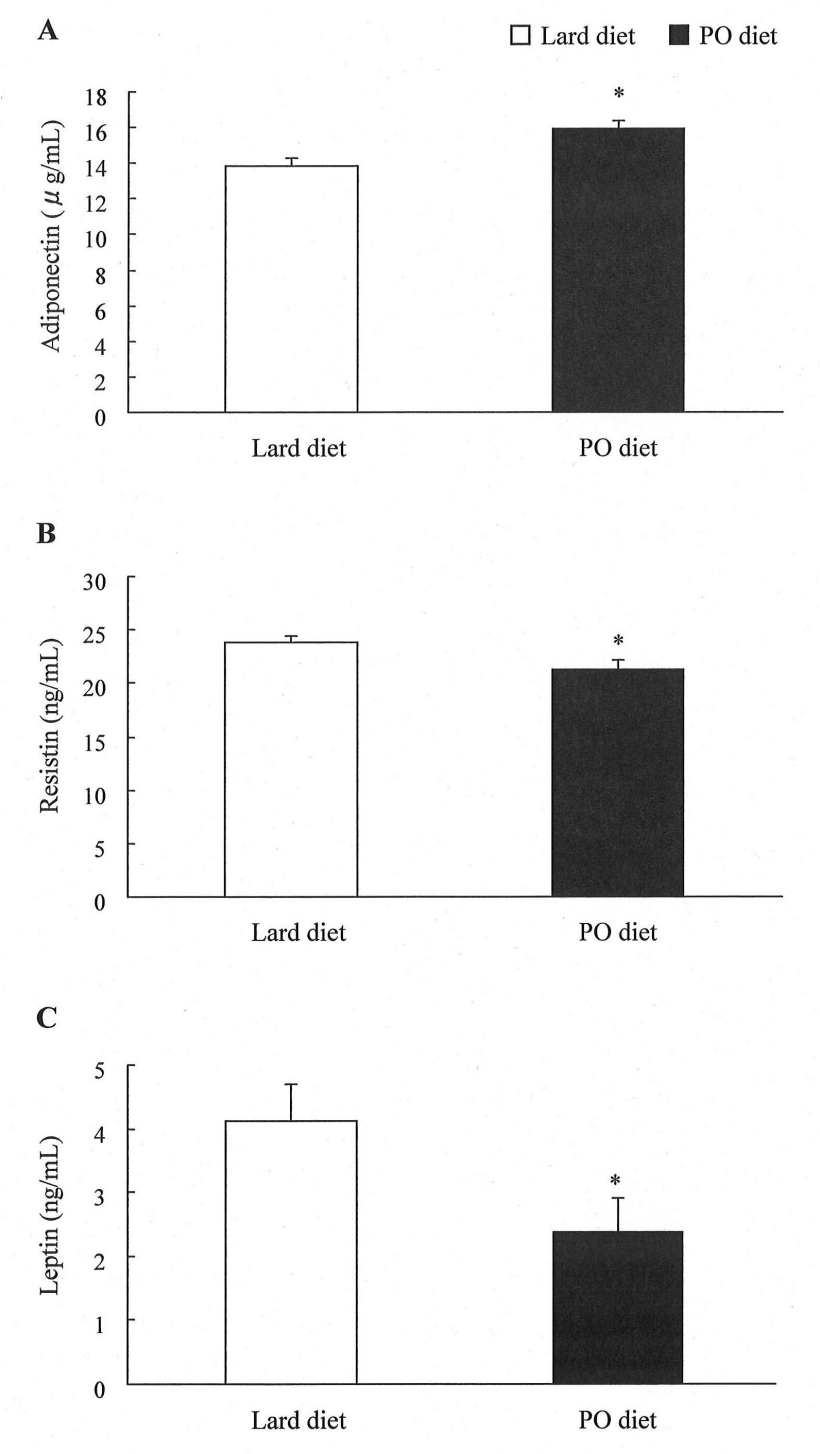
**Effect of pollock oil on plasma adipokine levels in mice fed a high-fat diet**. Plasma levels of adiponectin (A), resistin (B), and leptin (C) in C57BL/6J mice fed for 6 weeks with a diet containing 32% lard (lard diet) or 17% lard plus 15% pollock oil (PO diet). Values are mean ± SE (n = 10). *P < 0.05.

To determine if pollock oil could relieve the adipokine dysregulation caused by excessive total caloric intake, we measured the adipokine plasma levels. Intake of pollock oil increased the plasma concentration of adiponectin by 15% (*P *< 0.05) in the experimental group compared to the control group (Figure 1A). Plasma resistin and leptin concentrations were reduced by 14% (*P *< 0.05) and 41% (*P *< 0.05), respectively (Figure 1B and 1C).

### Effect of pollock oil on hepatic steatosis

**Figure 2:**
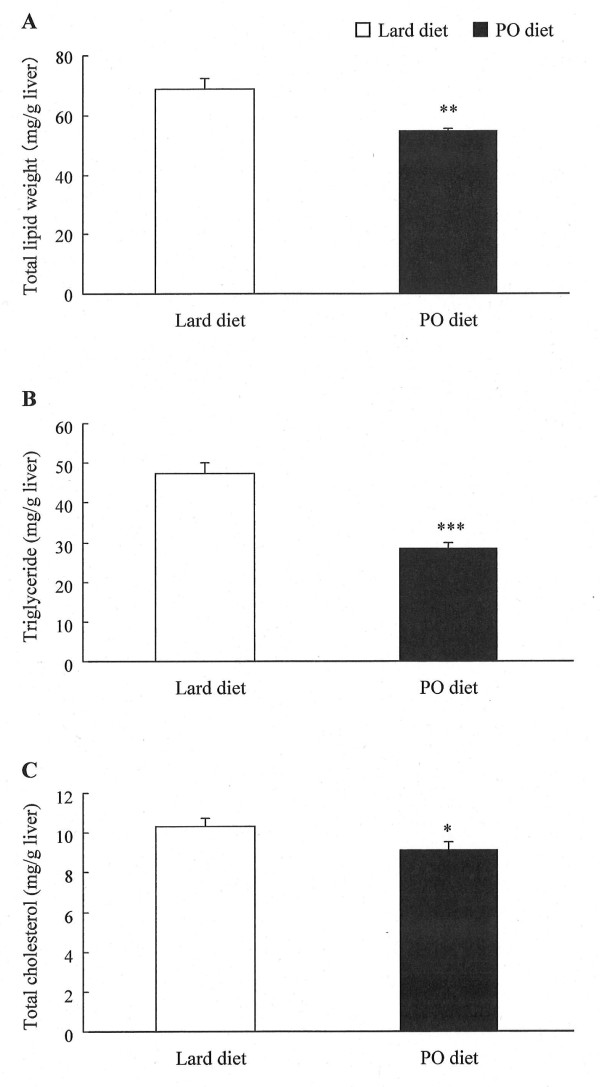
**Effect of pollock oil on hepatic steatosis in mice fed a high-fat diet**. Total hepatic lipid (A), triglyceride (B), and total cholesterol (C) levels in C57BL/6J mice fed for 6 weeks with a diet containing 32% lard (lard diet) or 17% lard plus 15% pollock oil (PO diet). Values are mean ± SE (n = 10). *P < 0.05; **P < 0.01; ***P < 0.001.

Because obesity can trigger hepatic steatosis, which is associated with hyperlipidemia, we measured the levels of hepatic lipids to determine if pollock oil suppressed hepatic lipid accumulation. Total hepatic lipid, triglyceride, and total cholesterol levels were reduced by 21% (*P *< 0.01), 40% (*P *< 0.001), and 12% (*P *< 0.05), respectively, in the experimental group compared with the control group (Figure 2).

### Effect of pollock oil on mRNA expression of genes involved in hepatic lipid metabolism

**Figure 3:**
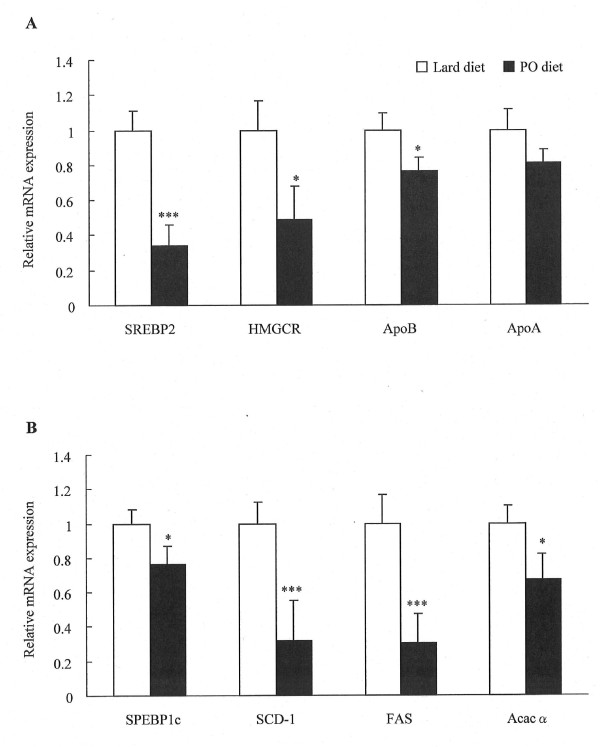
**Effect of pollock oil on transcription of genes related to hepatic cholesterol and lipid metabolism**. mRNA levels for genes involved in cholesterol (A) and lipid (B) metabolism in C57BL/6J mice fed for 6 weeks with a diet containing 32% lard (lard diet) or 17% lard plus 15% pollock oil (PO diet). Values are mean ± SE (n = 10). *P < 0.05; ***P < 0.001.

The pollock oil-supplemented diet led to a 66% reduction (*P *< 0.001) in mRNA expression of *SREBP2*, a gene that encodes a transcription factor involved mainly in regulation of cholesterol synthesis (Figure 3A). It also reduced expression of the cholesterogenic gene *HMGCR *by 51% (*P *< 0.05) and of *ApoB *by 23% (*P *< 0.05), although it had no significant effect on expression of *ApoA*. Furthermore, intake of pollock oil caused a 24% (*P *< 0.05) reduction in mRNA expression of *SREBP1c*, which encodes for the lipogenic transcription factor SREBP1c, and also reduced expression of the downstream lipogenic genes *SCD-1*, *FAS*, and *Acac*α by 68% (*P *< 0.001), 69% (*P *< 0.001), and 33% (*P *< 0.05), respectively (Figure 3B).

## Discussion

People with obesity have an increased risk of cardiovascular disease, which is a major cause of their increased mortality. Although multiple factors are thought to contribute to these elevated risks, one main determinant is the adverse effect of obesity on lipoprotein levels [10]. For the study reported herein, we examined the effect of pollock oil on dyslipidemia in diet-induced obese mice, and showed that inclusion of pollock oil in a high-fat diet significantly decreased plasma levels of total and LDL cholesterol and triglyceride.

To identify possible mechanisms underlying this reduction, we measured plasma adipokine levels. Adiponectin, an adipocyte-derived hormone, is believed to play an important role in regulating hyperglycemia, hyperlipidemia, and endothelial dysfunction in humans, all of which probably contribute to certain markedly increased risks associated with obesity-related disorders, e.g., atherosclerosis and diabetes [11]. Adiponectin levels correlate negatively with serum triglyceride levels in non-diabetic subjects and type-2 diabetics, and hypoadiponectinemia is associated with smaller LDL particle size, indicating a link between adiponectin and dyslipidemia [12-15]. Furthermore, *in vitro *data indicate that adiponectin is an anti-inflammatory and anti-proliferative mediator that can modulate atherosclerosis progression [16-18]. Conversely, increased levels of proinflammatory adipokines (e.g., tumor necrosis factor α, resistin, monocyte chemotactic protein 1, and interleukin 8) have been associated with increased serum lipids (e.g., triglycerides, and total and LDL cholesterol), which increase monocyte recruitment and adherence to arterial walls, causing wall remodeling [19-22]. In addition, leptin, the first adipocytokine discovered, has been associated with development of obesity, as *ob*/*ob *leptin-deficient mice are markedly obese [23]. However, most obese humans have increased blood leptin concentrations, likely reflecting resistance to the action(s) of leptin [24]. Leptin is also associated with increased insulin resistance, which can cause hyperlipidemia [25]. Therefore, the decreased plasma lipid concentrations observed following pollock oil ingestion were possibly associated with elevated plasma adiponectin levels and reduced plasma proinflammatory adipokine levels. As endogenous ligands for peroxisome proliferator-activated receptors, n-3 PUFAs regulate the expression of genes encoding key proteins involved in metabolism [26,27]. Intake of fish oil enriched in n-3 PUFAs has been reported to increase plasma adiponectin levels and lower proinflammatory adipokine levels [28,29]. Furthermore, we have shown that a diet rich in long-chain MUFAs modulates adipokine profiles [5]. Fatty acid composition analyses revealed that pollock oil ingestion significantly increased long-chain MUFA and n-3 PUFA levels in plasma and vital organs. Therefore, the favorable changes in the plasma adipokine profile might be attributed to a combined effect of n-3 PUFAs and long-chain MUFAs found abundantly in pollock oil.

Ingestion of pollock oil also decreased hepatic lipid levels in the experimental group, indicating that pollock oil inhibited hepatic steatosis triggered by obesity. The liver is the most important organ in energy metabolism, and is vital to the production and catabolism of plasma lipoproteins and endogenous lipids [30,31]. The improvement in fatty filtration in the livers of pollock oil-fed mice was possibly related to the decreased plasma lipid levels. To characterize how pollock oil decreased hepatic lipid accumulation, we also determined the hepatic mRNA levels of genes related to lipid metabolism. In the liver, sterol regulatory element-binding proteins (SREBPs) are key transcription factors that regulate the levels of lipids produced for export into the plasma as lipoproteins and into the bile as micelles. The SREBP family includes SREBP1a, 1c, and 2 [32]. SREBP1c regulates transcription of genes involved in fatty acid metabolism (e.g., *SCD-1*, *FAS*, and *Acac*α), and SREBP2 regulates transcription of cholesterol-related genes (e.g., *HMGCR*, which encodes the rate-limiting enzyme of cholesterol biosynthesis). Increased SREBP activity causes cholesterol and fatty acid accumulation and downregulates the SREBP-cleavage-activating protein (SCAP)/SREBP pathway by feedback inhibition [33]. Our data show that, concomitant with downregulation of SREBP1c and SREBP2 mRNA, intake of pollock oil suppressed transcription of genes targeted by SREBP. Furthermore, pollock oil intake inhibited hepatic *ApoB *transcription. Apolipoprotein B is central in lipoprotein metabolism [34], serving as a structural and functional component of triglyceride-rich very-low-density lipoproteins and their metabolic products, e.g., intermediate-density lipoprotein and LDL. Downregulation of *ApoB *expression may therefore decrease circulating LDL cholesterol levels. Notably, pollock oil ingestion did not alter mRNA expression of *ApoA*, the structural component of HDL, which may reflect the fact that plasma HDL cholesterol levels were the same in the control and experimental groups. Collectively, the observed decreases in mRNA levels suggest that the improvements in hepatic lipid levels and associated hyperlipidemia found in the experimental group may be partially associated with decreased *de novo *cholesterol, lipid, and ApoB synthesis.

## Conclusions

Our study showed that ingestion of pollock oil ameliorated hypercholesterolemia and hypertriacylglyceridemia in diet-induced obese mice. The hypolipidemic effect of pollock oil was possibly related to an increase in plasma adiponectin concentrations and a decrease in the plasma levels of proinflammatory adipokines. Downregulation of mRNA expression of lipogenic genes and genes involved in cholesterol metabolism positively affected hepatic lipid accumulation, which likely led to an improved plasma lipid profile.

## List of abbreviations

Acacα: acetyl-coenzyme A carboxylase alpha; ApoA: apolipoprotein A; ApoB: apolipoprotein B; FAS: fatty acid synthase; HDL: high-density lipoprotein; HMGCR: 3-hydroxy-3-methylglutaryl-coenzyme A reductase; LDL: low-density lipoprotein; MUFA: monounsaturated fatty acids; PUFA: polyunsaturated fatty acids; QPCR: quantitative polymerase chain reaction; SCD-1: stearoyl-coenzyme A desaturase-1; SREBP: sterol regulatory element binding protein; WAT: white adipose tissue.

## Competing interests

The authors declare that they have no competing interests.

## Authors' contributions

ZHY participated in the planning of the study, data analysis, and manuscript preparation. HM participated in experimental work. JT, AH and MK participated in the planning and organization of the study. All authors read and approved the final manuscript.
